# Recognition of specific sialoglycan structures by oral streptococci impacts the severity of endocardial infection

**DOI:** 10.1371/journal.ppat.1007896

**Published:** 2019-06-24

**Authors:** Barbara A. Bensing, Liang Li, Olga Yakovenko, Maurice Wong, Karen N. Barnard, T. M. Iverson, Carlito B. Lebrilla, Colin R. Parrish, Wendy E. Thomas, Yan Xiong, Paul M. Sullam

**Affiliations:** 1 Department of Medicine, San Francisco Veterans Affairs Medical Center and University of California, San Francisco, California, United States of America; 2 Los Angeles Biomedical Research Institute, Harbor-UCLA Medical Center, Torrance, California, United States of America; 3 Department of Bioengineering, University of Washington, Seattle, Washington, United States of America; 4 Department of Chemistry, University of California, Davis, California, United States of America; 5 Baker Institute for Animal Health, Department of Microbiology and Immunology, College of Veterinary Medicine, Cornell University, Ithaca, New York, United States of America; 6 Departments of Pharmacology and Biochemistry, Vanderbilt University, Nashville, Tennessee, United States of America; 7 David Geffen School of Medicine at UCLA, Los Angeles, California, United States of America; Boston Children's Hospital, UNITED STATES

## Abstract

*Streptococcus gordonii* and *Streptococcus sanguinis* are primary colonizers of the tooth surface. Although generally non-pathogenic in the oral environment, they are a frequent cause of infective endocarditis. Both streptococcal species express a serine-rich repeat surface adhesin that mediates attachment to sialylated glycans on mucin-like glycoproteins, but the specific sialoglycan structures recognized can vary from strain to strain. Previous studies have shown that sialoglycan binding is clearly important for aortic valve infections caused by some *S*. *gordonii*, but this process did not contribute to the virulence of a strain of *S*. *sanguinis*. However, these streptococci can bind to different subsets of sialoglycan structures. Here we generated isogenic strains of *S*. *gordonii* that differ only in the type and range of sialoglycan structures to which they adhere and examined whether this rendered them more or less virulent in a rat model of endocarditis. The findings indicate that the recognition of specific sialoglycans can either enhance or diminish pathogenicity. Binding to sialyllactosamine reduces the initial colonization of mechanically-damaged aortic valves, whereas binding to the closely-related trisaccharide sialyl T-antigen promotes higher bacterial densities in valve tissue 72 hours later. A surprising finding was that the initial attachment of streptococci to aortic valves was inversely proportional to the affinity of each strain for platelets, suggesting that binding to platelets circulating in the blood may divert bacteria away from the endocardial surface. Importantly, we found that human and rat platelet GPIbα (the major receptor for *S*. *gordonii* and *S*. *sanguinis* on platelets) display similar O-glycan structures, comprised mainly of a di-sialylated core 2 hexasaccharide, although the rat GPIbα has a more heterogenous composition of modified sialic acids. The combined results suggest that streptococcal interaction with a minor O-glycan on GPIbα may be more important than the over-all affinity for GPIbα for pathogenic effects.

## Author summary

Infective endocarditis (IE) is a life-threatening infection of heart valves, and streptococci that normally reside in the mouth are a leading cause of this disease. Some oral streptococcal species express a protein on their surface that enables attachment to glycan (sugar) modifications on saliva proteins, an interaction that may be important for colonization of the tooth and other oral surfaces. These "Siglec-like adhesins" are hypervariable in the type and number of glycan structures they bind, ranging from just one to more than six of the structures displayed on the saliva proteins. If streptococci enter into the bloodstream, the Siglec-like adhesin can mediate attachment to similar glycans that decorate platelet or plasma proteins, which can impact the overall virulence of the organism. This study highlights how recognition of a specific type of glycan structure can cause a generally beneficial or neutral microbe to create damage to specific tissues—in this case the heart valves, illustrating one means by which commensal bacteria can become opportunistic or accidental pathogens. The findings further indicate that certain glycan-binding streptococci among the oral microbiota may be predisposed to produce infective endocarditis.

## Introduction

Infective endocarditis (IE) is a life-threatening cardiovascular disease in which microbes colonize and persist in platelet-fibrin thrombi on cardiac valve surfaces. The interaction of bacteria with platelets is thought to play a central role in the pathogenesis of IE [1, 2]. Most bacterial species are unable to colonize an intact cardiac valve endothelium, but instead attach to platelet-fibrin thrombi or "sterile vegetations" that have deposited on damaged valve surfaces [3–5]. The subsequent deposition of platelets onto the infected endocardium, along with bacterial proliferation, contributes to the progression of disease, and results in the formation of macroscopic endocardial lesions [6–8].

**Fig 1:**
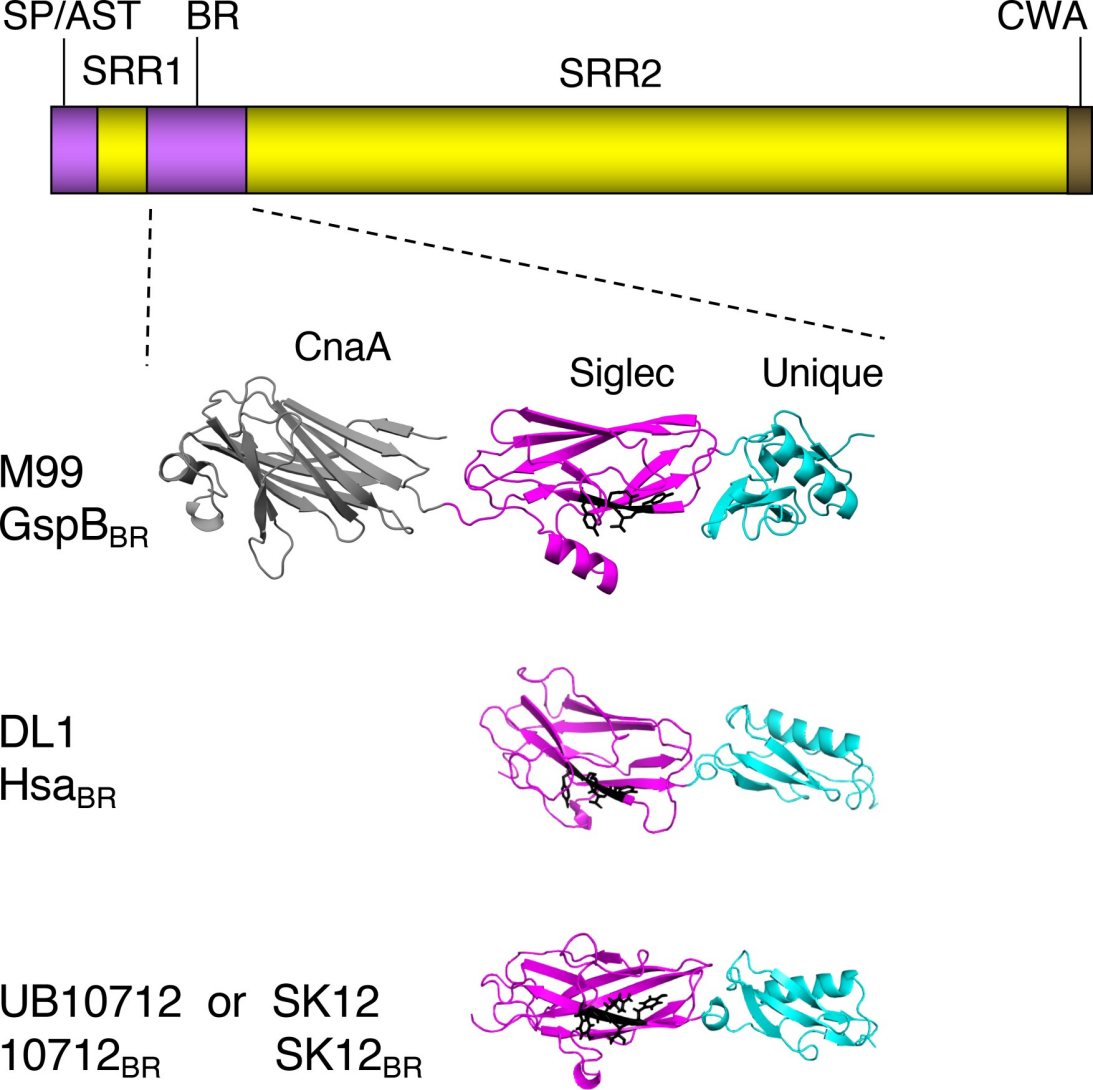
Comparison of the GspB, Hsa and 10712 BRs. The upper diagram shows the general domain organization of the SRR glycoproteins. SP/AST, signal peptide and accessory Sec transport domain; BR, ligand binding region; CWA, cell wall anchor. The SRR1 and SRR2 regions undergo glycosylation in the bacterial cytoplasm, prior to transport by the accessory Sec system. The lower portion shows high-resolution crystal structures of the binding regions of GspB, Hsa and the SRR glycoprotein from the *S*. *gordonii* strain UB10712. The GspB_BR_ structure was reported previously [30]. Partially refined structures of the Hsa_BR_ and 10712_BR_ were provided by T. Iverson (manuscript submitted; PDB files 6EFC and 6EFF pending release). Note that the GenBank entry for the 10712_BR_ sequence originally listed the source strain as *Streptococcus mitis* NCTC10712 (GenBank JYGN00000000) [66]. The *S*. *gordonii* SK12 BR sequence is identical to that of UB10712, and was obtained by translation of the publicly available partially assembled SK12 genome (NZ_LAWP01000015). The CnaA domain is found in some Siglec-like BRs but does not contribute to sialoglycan binding. The sialoglycan ligand preferences thus far appear to be dictated by the Siglec domains. The YTRY motif residues are shown as black sticks. The Unique domain may modulate the conformation of the Siglec domain.

*Streptococcus gordonii* and *Streptococcus sanguinis* are oral commensal bacterial species that are primary colonizers of tooth surfaces [9]. Although generally associated with oral health, these closely-related species are frequently found as the causative agent of infective endocarditis, especially infections of the aortic valve [10–14]. Only a small number of virulence factors of *S*. *sanguinis* or *S*. *gordonii* that contribute to IE have been verified using animal models of this disease [15–22]. Among the best characterized for *S*. *gordonii* are the platelet-binding proteins GspB and Hsa, expressed by strains M99 and DL1, respectively. These cell wall anchored adhesins are two members of the highly-conserved family of serine-rich repeat (SRR) glycoproteins expressed by Gram-positive bacteria (Fig 1). The ligand-binding regions (BRs) of the SRR glycoproteins are modular and often species-specific [23, 24]. SRR glycoprotein sequences have been found in the genomes of all *S*. *sanguinis* and *S*. *gordonii* strains sequenced to date [25], and invariably contain "Siglec-like" BRs that confer high-affinity binding to α2–3 linked sialic acid [23, 26]. This sialoglycan modification is displayed at the termini of O-glycans that decorate the salivary mucin MUC7 [27, 28], and binding of *S*. *gordonii* and *S*. *sanguinis* to MUC7 is thought to be important for oral colonization. In addition, previous studies indicate that when oral streptococci enter the bloodstream, binding to similar O-glycans on platelet GPIbα (the receptor for von Willebrand factor, or vWF) can contribute to the pathogenesis of IE [29, 30].

**Fig 2:**
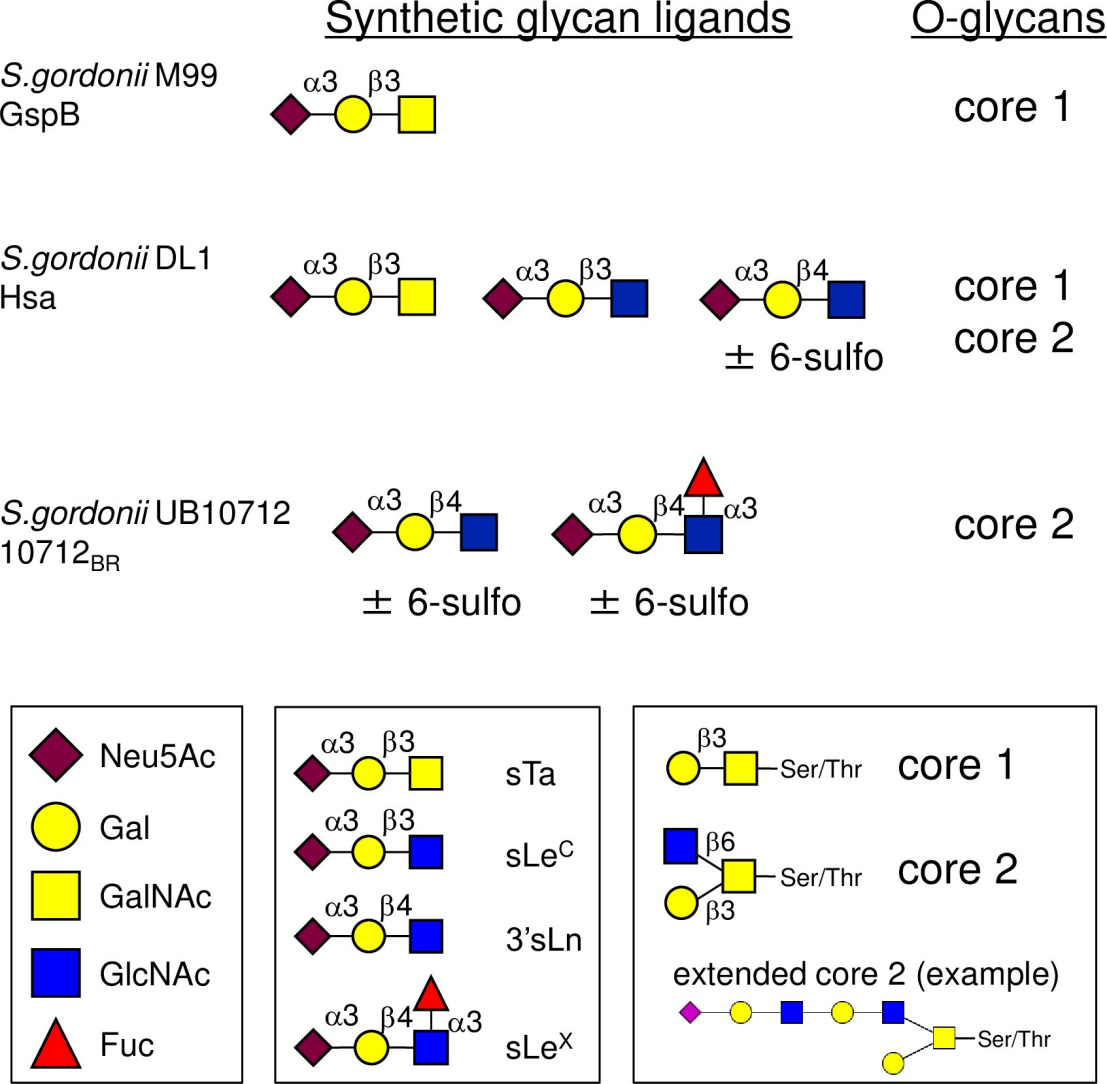
Ligand repertoires determined for three Siglec-like BRs. The high affinity sialoglycan ligands for the recombinant BRs were determined by analysis on a custom sialoglycan array and by enzyme-linked assays [23, 26]. The O-glycan ligand preferences were determined by analyzing glycan profiles of affinity-captured plasma proteins [33]. Note that 10712_BR_ was previously referred to as NCTC10712_BR_. Binding of strains M99 and DL1 to array glycans paralleled binding of the respective recombinant BRs [26]. Monosaccharide symbols follow the Symbol Nomenclature for Glycans system [67]. Neu5Ac, N-acetyl neuraminic acid; Gal, galactose; GalNAc, N-acetyl galactosamine; GlcNAc, N-acetyl glucosamine; Fuc, fucose. sTa, sialyl T-antigen; sLe^C^, sialyl Lewis C; 3'sLn, 3'sialyllactosamine; sLe^X^, sialyl Lewis X. Throughout the manuscript, 3'sLn is referred to simply as "sLn". The "core 1" and "core 2" designations refer to the protein-proximal glycan structures.

The Siglec-like BRs are an intriguing group of hypervariable adhesive domains, displaying both conserved and divergent features (Fig 1). They all contain Siglec and Unique domains that are important for sialoglycan binding [23, 26, 30–32]. The BRs of some *S*. *sanguinis* and most *S*. *gordonii* SRR adhesins, such as GspB, also include a CnaA domain, but this region appears not to have a role in sialoglycan binding [23]. The Siglec domain has a V-set Ig fold resembling that of mammalian Siglecs, and includes a conserved "YTRY motif" that makes important contacts with Neu5Acα2-3Gal at the termini of larger glycans [31, 32]. The Unique domain does not appear to make direct contacts with sialoglycans, but may modulate the conformation and thus influence the binding properties of the Siglec domain. Despite a conserved structural fold, the Siglec domain sequences can vary by more than 50%, and both small and large sequence variations can impact the number and type of sialoglycan structures recognized. Specific glycan targets have been identified for nearly a dozen of the Siglec-like BRs, and the ligand repertoires range from a single type of sialylated trisaccharide, to a broad set of related sialoglycans [23, 26]. For example, GspB is highly selective for sialyl T-antigen (sTa) [23, 26], whereas the 10712_BR_ (from the SRR adhesin of *S*. *gordonii* UB10712) preferentially binds 3'sialyllactosamine (sLn; Fig 2) [23]. Hsa has a broader ligand range and can bind both sTa and sLn [23, 26]. The differences in binding to defined, synthetic glycans are also reflected in the interaction with O-glycosylated plasma proteins [33]. GspB most readily binds proteins bearing sTa (a core 1 O-glycan; Fig 2), while 10712_BR_ prefers proteins with sLn at the termini of larger, branched, and often extended core 2 glycans. The ligand repertoire also impacts the strength of binding of the recombinant BRs to platelet GPIbα, with binding to sLn generally conferring a higher affinity for platelets and GPIbα compared with binding to sTa [23, 33]. As measured by surface plasmon resonance, the affinity of recombinant GspB_BR_ for GPIbα is 2.38 × 10^−8^ M, whereas Hsa_BR_ has approximately 5-fold higher affinity (K_D_ values of 3.05 × 10^−8^ M and 5.05 × 10^−9^ M when fit to a heterogenous ligand model, which is consistent with the ability to bind two glycan moieties) [29].

The role of the Siglec-like adhesins and sialoglycan binding in streptococcal endocarditis is not entirely clear. Deletion of *gspB* or *hsa* from *S*. *gordonii* strains, or even just a single amino acid substitution in the YTRY motif of GspB (GspB^R484E^), resulted in two-log lower levels of bacteria in aortic valve vegetations using a rat model of IE [15, 16, 30]. In contrast, deletion of *srpA* did not adversely impact the virulence of *S*. *sanguinis* SK36 in a rabbit model of IE [21]. Although the precise sialoglycan ligand for SrpA has not been determined, it does not readily bind sTa, but instead may recognize a core 2 hexasaccharide or larger di-sialylated O-glycan [32, 33]. Supporting the possibility that the type of sialoglycan recognized might influence disease progression, *S*. *gordonii* strain SK12 was found to be significantly less virulent than *S*. *gordonii* DL1 in a rat model of IE [34]. SK12 encodes an SRR glycoprotein with a BR identical to that of the 10712_BR_ (see legend to Fig 1), and thus is likely to bind sLn rather than sTa. Additional analysis of Siglec-like BRs from a small number of streptococcal strains suggested that IE and commensal strains might bind different glycan structures, in that IE isolates were more often GspB-like, whereas oral isolates were more SrpA-like [23, 25]. However, the question of whether binding to a particular sialoglycan structure, versus sialic acid binding in general, affects the propensity of bacteria to establish endovascular infections has never been formally assessed. In this study, we generated a set of isogenic strains that display distinctly different sialoglycan binding properties and different levels of binding to platelets. We then compare the relative virulence of these strains in two rat models of IE. The results indicate that the sialoglycan binding spectrum can impact the overall virulence of streptococci, displaying different effects on the initial colonization of aortic valves, as well as the post-colonization progression of endocardial infection.

## Results

### Platelet and sialoglycan binding by isogenic variant strains

**Fig 3:**
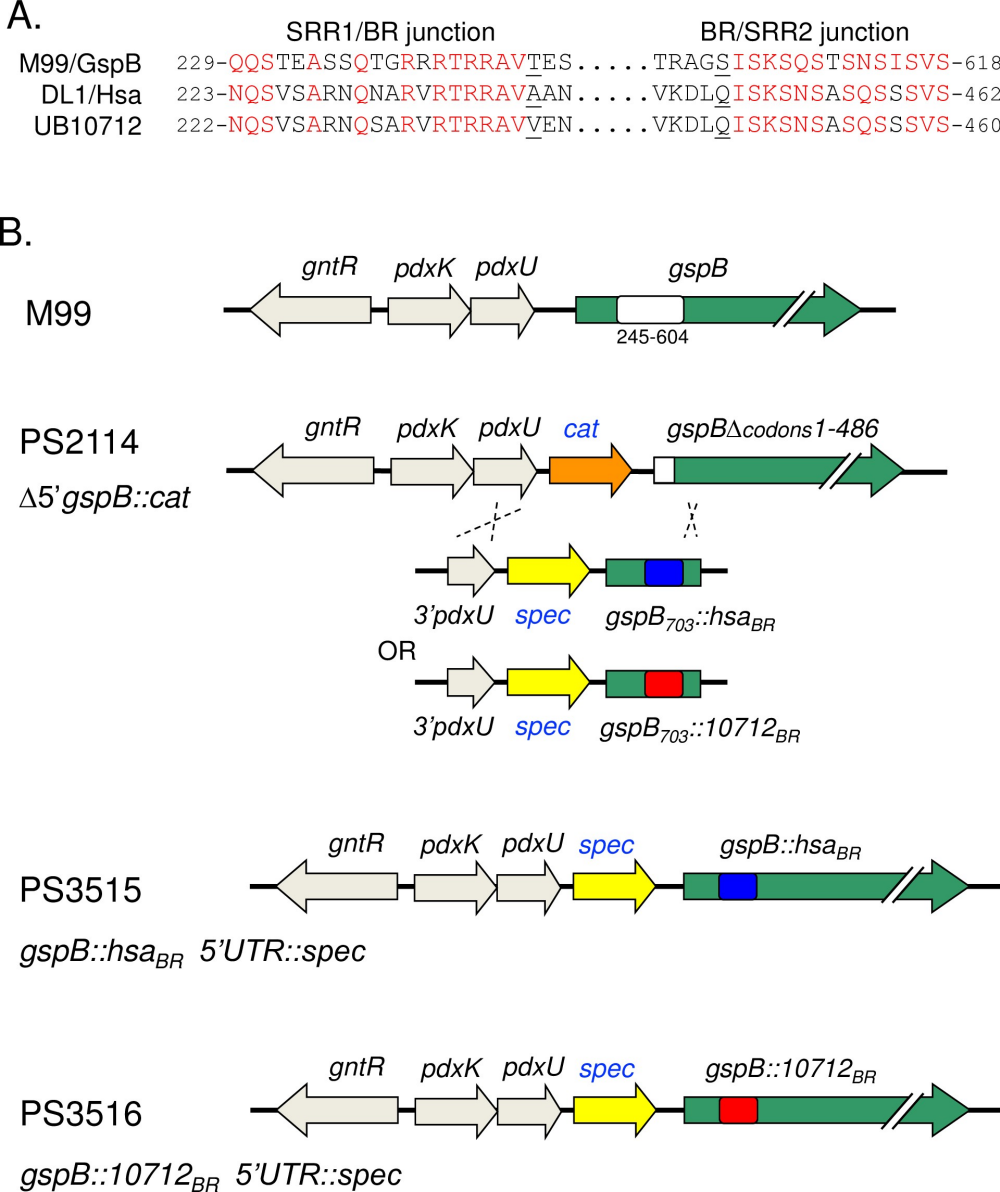
Design of isogenic variant strains of *S*. *gordonii* M99. Strains were designed to express GspB variants in which amino acid residues 248 to 604 of GspB (GenBank accession number AAL13053) were replaced with residues 242 to 448 of Hsa (ABV10391) or 241 to 446 of the SRR glycoprotein homolog from *S*. *gordonii* UB10712 (WP_045635027). A: Alignment of the BR domain junctions. Conserved amino acids are indicated in red type. T248 and S604 of GspB, A242 and Q448 of Hsa, and V241 and Q446 of the UB10712 homolog are underlined. B: Strategy to replace the GspB BR coding region in the native *S*. *gordonii* chromosomal locus. PS2114 is a derivative of *S*. *gordonii* M99 that has a deletion of *gspB* codons 1 to 486 and a *cat* gene in the upstream non-coding region [30]. Chimeric sequences were introduced into the *S*. *gordonii* chromosome via a strategy that involved recombination by double-crossover between *gspB* codons 605–703 and the upstream *pdxU* gene.

**Fig 4:**
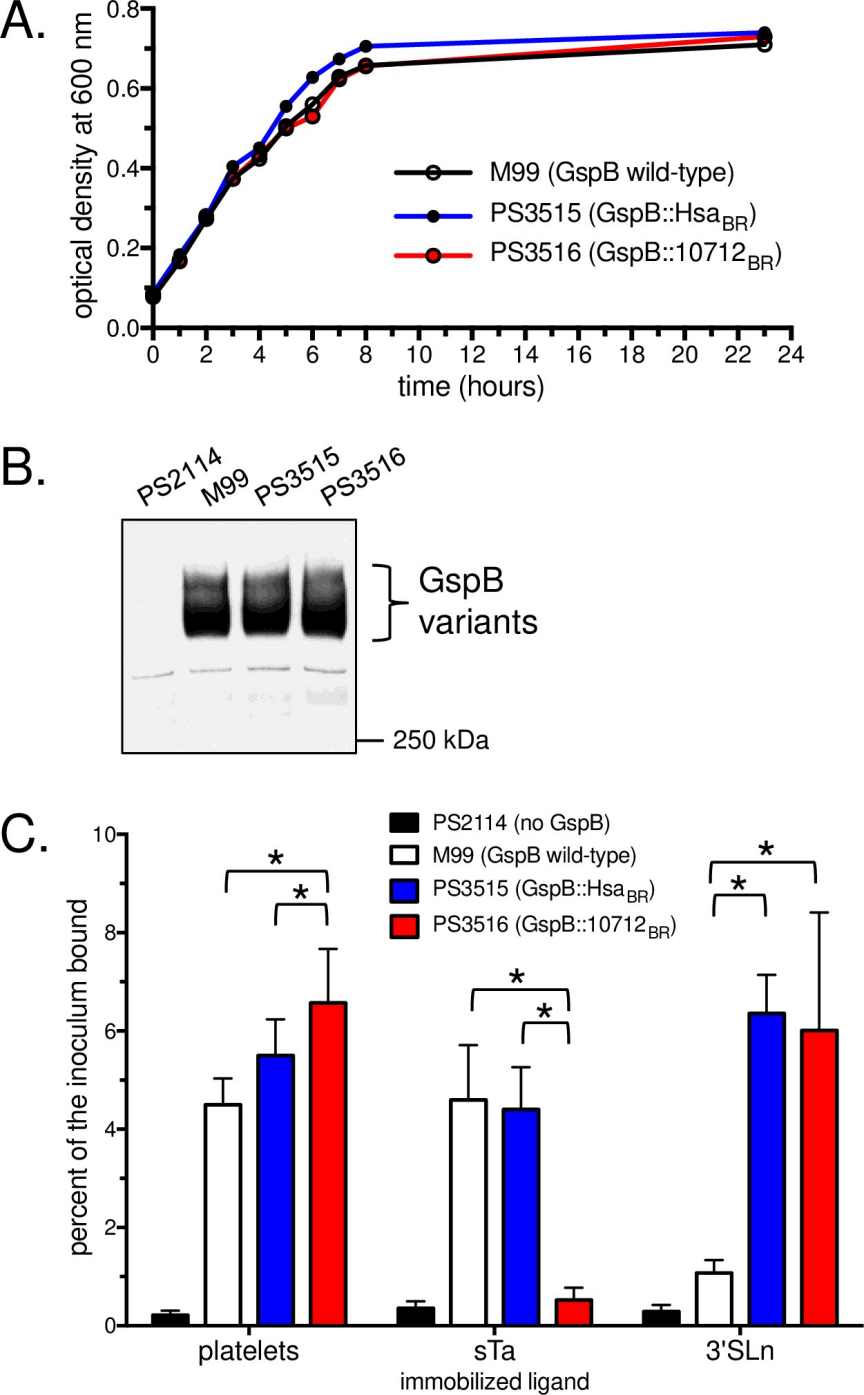
Characteristics of the chimeric SRR glycoprotein *S*. *gordonii* strains. A: The isogenic variant strains show growth rates similar to M99. Strains were grown for 17 h in Todd-Hewitt broth, then diluted 1:10 into fresh medium and incubated at 37°C for 23 h. B: The chimeric SRR glycoproteins display normal cell-surface expression levels and apparent molecular masses. Each lane contains cell wall proteins extracted from bacteria in 75 μl of stationary-phase cultures (roughly 75 x 10^6^ CFU). Blots were probed with polyclonal antibodies that recognized the glycan moieties on GspB. C: Binding properties of the isogenic variant strains. Fixed human platelets or biotinylated glycans were immobilized in 96-well plates. Binding is reported as the mean ± standard deviation of the percent of input streptococci adherent after 90 min (n = 6). Asterisks indicate p<0.05.

Our first goal was to generate isogenic variants of *S*. *gordonii* strain M99 that differ in their sialoglycan binding phenotypes. We selected three BRs that were previously determined to have distinctly different binding properties (Fig 2): 1) GspB_BR_ demonstrates sTa selectivity (core 1 O-glycans), 2) the 10712_BR_ has high affinity for sLn and related structures (core 2 O-glycans), and 3) Hsa_BR_ shows high-affinity binding to both sTa and sLn (core 1 and core 2 glycans). The design of these isogenic strains was not trivial, since SRR glycoprotein expression relies on a complex and highly specialized system that coordinates post-translational modification and transport to the bacterial cell surface. For example, in *S*. *gordonii* M99 and *Streptococcus parasanguinis* FW12, elements in the preprotein mature region, as well as the N-terminal signal peptide, must be matched to the dedicated SecA2/Y2 transporter [35–38]. It was also important to avoid any alterations in the flanking SRR regions, since the post-translational modification of these domains can impact binding [39–43]. In view of these issues, we chose to replace the entire BR of GspB with that of Hsa, or with the 10712_BR_, using the conserved SRR1-BR and BR-SRR2 junctions (Fig 3A), while retaining the native GspB signal peptide, AST, SRR1 and SRR2 domains. To ensure native expression levels *in vivo* we opted to replace a portion of the *gspB* gene in the native chromosomal locus, using a "knock in" strategy previously used to generate single amino acid substitutions in the YTRY motif of the Siglec domain (Fig 3B). This resulted in strains PS3515 (GspB::Hsa_BR_) and PS3516 (GspB::10712_BR_). Importantly, the variant strains showed growth rates and cell-surface SRR glycoprotein expression levels (i.e. SDS migration patterns and western blot intensity) that were indistinguishable from the parental M99 strain (Figs 4A and 4B and S1).

We next examined bacterial binding to synthetic sialoglycans or to immobilized human platelets. The binding of these strains to sialoglycans resembled that of the respective recombinant BRs: M99 readily bound to sTa but not sLn, PS3515 bound both sialoglycan structures, and PS3516 bound sLn rather than sTa (Fig 4C). Likewise, strain PS3516 showed higher levels of binding to platelets as compared with the parental strain M99 or with PS3515 (p = 0.0001 or 0.0397, respectively; Fig 4C), paralleling what was previously reported for the recombinant BRs [23, 31, 33]. Thus, the isogenic strains display the anticipated sialoglycan binding specificities.

### Impact of sialoglycan binding on streptococcal endocarditis

**Fig 5:**
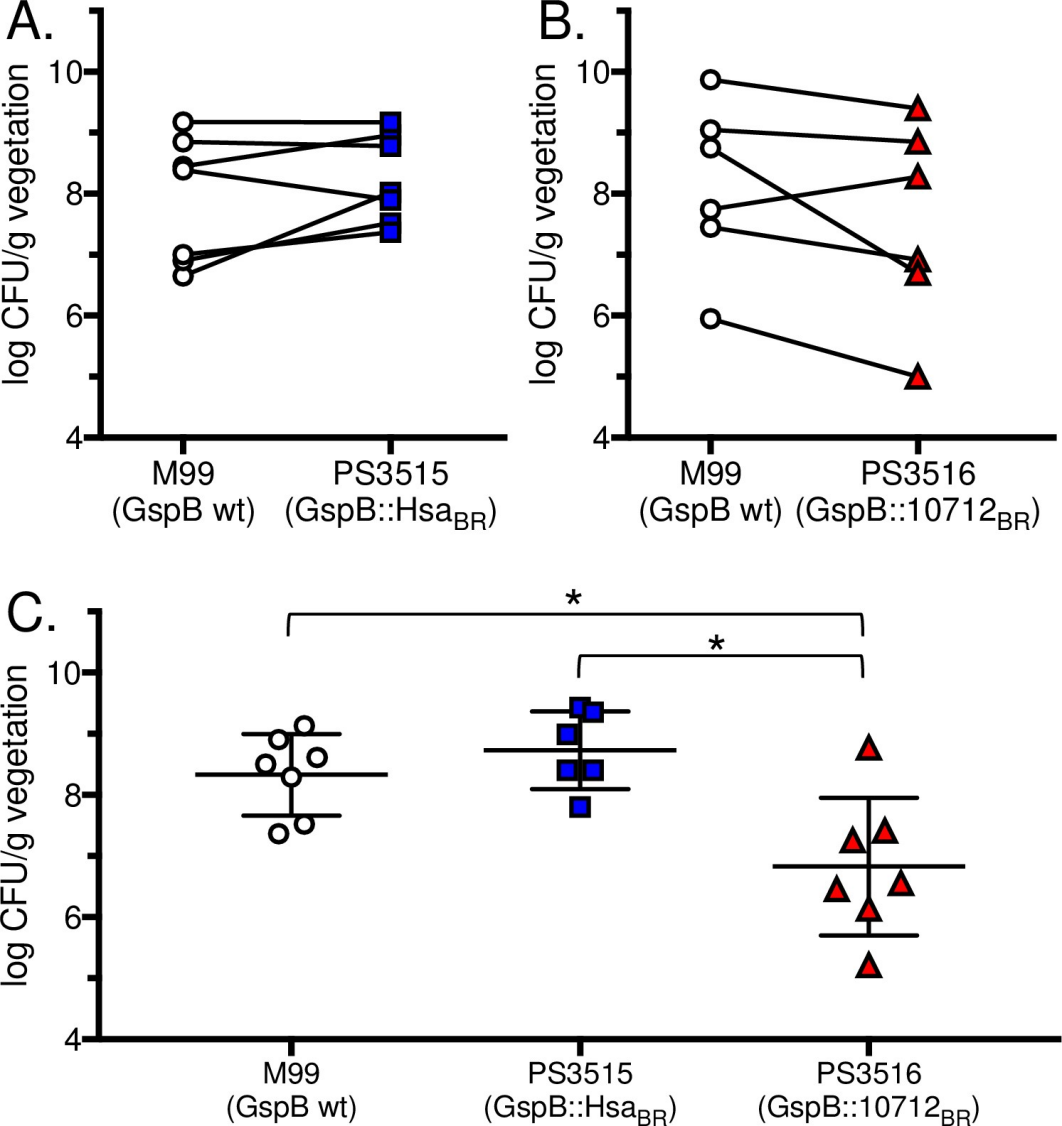
Relative virulence of *S*.*gordonii* M99 and the isogenic variant strains in two rat models of endocarditis. Animals were infected with 10^5^ CFU of each of a pair of strains (A and B; n = 7 and n = 6, respectively) or with 10^5^ CFU of a single strain (C; n = 7 for M99, n = 6 for PS3515 and n = 7 for PS3516). The number of bacteria in aortic valve vegetations was assessed 72 h post-infection. Asterisks indicate p<0.05.

To assess the impact of binding to sTa versus sLn on endocarditis, we used two versions of a well-established animal model for this disease. The first was a competition assay, in which rats were catheterized to induce aortic valve damage and platelet-fibrin deposition, and then infected intravenously with an inoculum containing 2 x 10^5^ CFU of M99 and an isogenic variant at a 1:1 ratio. At 72 h post-infection, animals were sacrificed and the relative number of each strain in aortic valve vegetations, kidneys and spleens were determined. Using this model, trends were apparent, with PS3515 showing higher average numbers in vegetations, kidney and spleen, and PS3516 showing lower densities compared with M99 (Fig 5A and 5B and Table 1). However, despite these trends (5 of 6 animals in the latter case) the differences were found not to reach statistical significance (p>0.05).

**Table 1:** Competition model of endocarditis in rats, 72 h post-infection.

Strains (Number of animals)	Log_10_ CFU/g tissue (mean ± SD)		
	Vegetation^a^	Kidney	Spleen
M99 (n = 7)	7.92 ± 1.04	3.12 ± 0.58	3.55 ± 1.04
PS3515 (n = 7)	8.25 ± 0.72	3.57 ± 1.10	3.92 ± 0.74
M99 (n = 6)	8.14 ± 1.39	2.99 ± 1.44	2.98 ± 1.42
PS3516 (n = 6)	7.52 ± 1.63	2.85 ± 1.23	2.73 ± 1.36

^a^ data are also shown in Fig 5A and 5B

We then used a second established model of IE, in which catheterized animals were infected intravenously with 10^5^ CFU of a single strain. At 72 h post-infection, animals infected with strain M99 or PS3515 had comparable levels (CFU/g) of bacteria within aortic valve vegetations (Fig 5C and Table 2). In contrast, rats infected with strain PS3516 had significantly lower densities of bacteria within aortic valve vegetations, when compared with either M99 or PS3515 (p = 0.011 and p = 0.002, respectively). Levels of bacteria within the kidneys of animals infected with strain M99 were significantly higher than in animals infected with either PS3515 or PS3516 (p = 0.049 and p = 0.001, respectively; Table 2). Importantly, no differences were seen in the number of bacteria in the blood or spleen 72 h post-infection (Table 2), indicating that the differences seen in the heart and kidney were not likely due to differences in the bacterial susceptibility to innate host defenses. These results indicate that the ability to bind sTa (M99 and PS3515) contributes to increased virulence, as measured by bacterial levels within aortic valve vegetations. In addition, selective binding to sTa (M99 versus PS3515 or PS3516) results in higher densities within kidneys, suggesting a greater tendency to disseminate from the heart to other organs.

**Table 2:** Single strain infection model of endocarditis in rats, 72 h post-infection.

Strain (Number of animals)	Log_10_ CFU/g tissue (mean ± SD)			Log_10_ CFU/ml (mean ± SD) Blood
	Vegetation^a^	Kidney	Spleen	
M99 (n = 7)	8.33 ± 0.67	5.04 ± 0.72^c^	3.62 ± 0.97	1.96 ± 0.92
PS3515 (n = 6)	8.73 ± 0.64	3.90 ± 0.53	3.53 ± 0.31	2.26 ± 0.48
PS3516 (n = 7)	6.83 ± 1.13^b^	3.12 ± 1.02	3.44 ± 0.47	2.21 ± 1.13

^a^ data are also shown in Fig 5C

^b^ p<0.05 compared with both M99 and PS3515

^c^ p<0.05 compared with both PS3515 and PS3516

### Impact of sialoglycan binding on colonization of aortic valves

**Fig 6:**
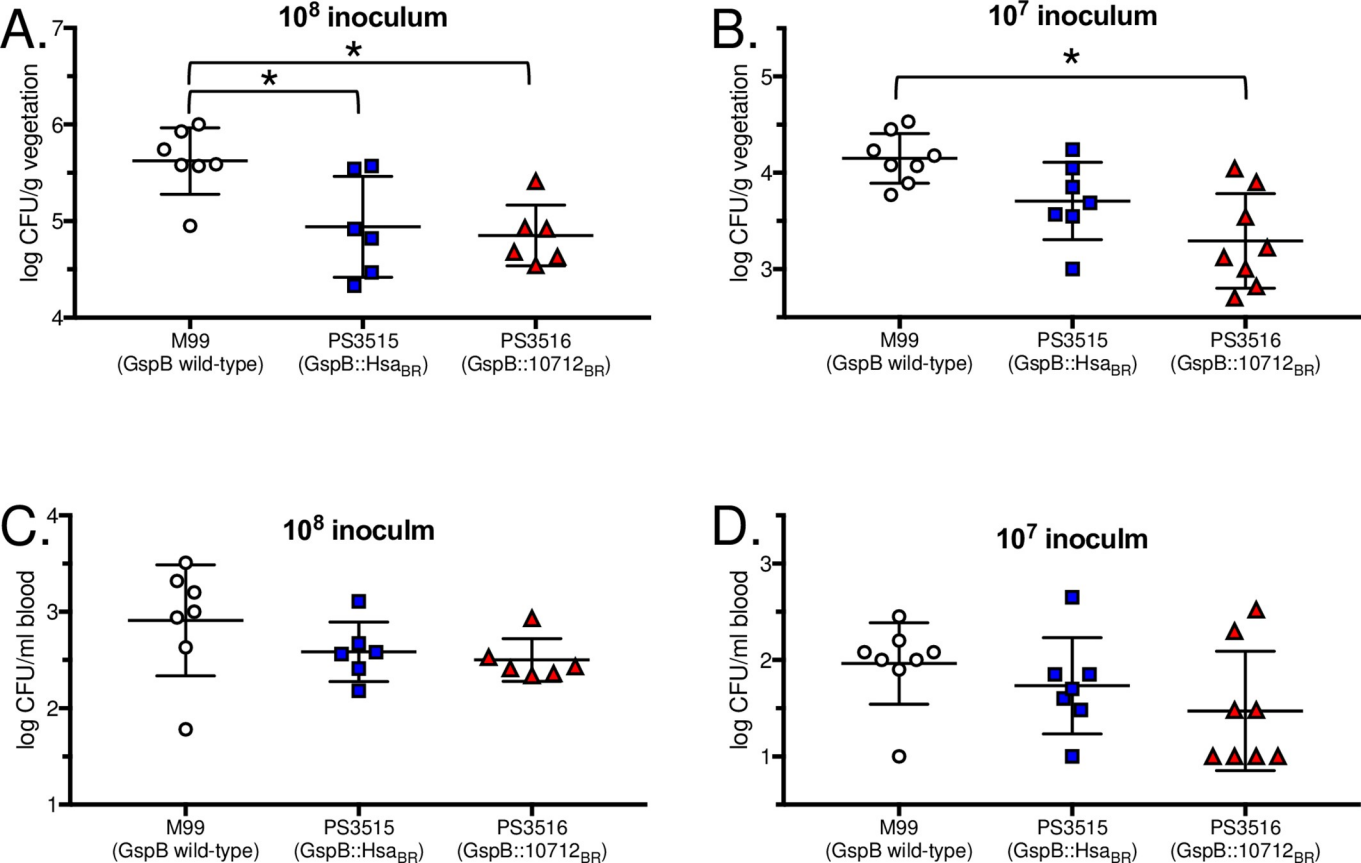
Initial colonization of mechanically-damaged aortic valves. Animals were infected with 10^8^ or 10^7^ CFU of the indicated strain. At the 10^8^ CFU inoculum, n = 7 for M99, and n = 6 for PS3515 and PS3516. For the 10^7^ CFU inoculum, n = 8 for M99 and PS3516, and n = 7 for PS3515. Numbers of bacteria attached to aortic valve vegetations (A and B) or in the peripheral blood (C and D) were assessed 1 h post-infection. Asterisks indicate p<0.05.

We next examined whether the differences in bacterial densities within aortic valve vegetations at 72 h post-infection were likely due to differences in the initial attachment of circulating bacteria to valve surfaces. Catheterized rats were infected intravenously with 10^8^ or 10^7^ CFU of M99, PS3515 or PS3516. At one hour after infection with 10^8^ CFU, rats given M99 had higher levels of bacteria on aortic valves, compared with either PS3515 or PS3516 (Fig 6A; p = 0.020 or 0.009, respectively). After infection with 10^7^ CFU, levels of PS3516 on valves were again significantly lower than those of M99 (p = 0.001). Levels of PS3515 were intermediate between those of M99 and PS3516, but not significantly different from either (Fig 6B). No significant differences were seen in the numbers of bacteria in the peripheral blood at either inoculum level (Fig 6C and 6D). These results indicate that binding of bacteria to sLn rather than sTa (PS3516 versus M99) results in diminished initial colonization. The lower extent of initial colonization does not fully account for the reduced numbers of PS3516 seen at 72 h, since the initial attachment of this strain was similar to that of PS3515 (Fig 6A and 6B), yet the latter showed two-log higher density in vegetations after 72 h (Fig 5C). Thus, the combined *in vivo* animal studies indicate that streptococcal binding to sTa contributes to higher bacterial densities subsequent to colonization of the damaged endocardium.

### Initial colonization is inversely proportional to platelet binding

**Fig 7:**
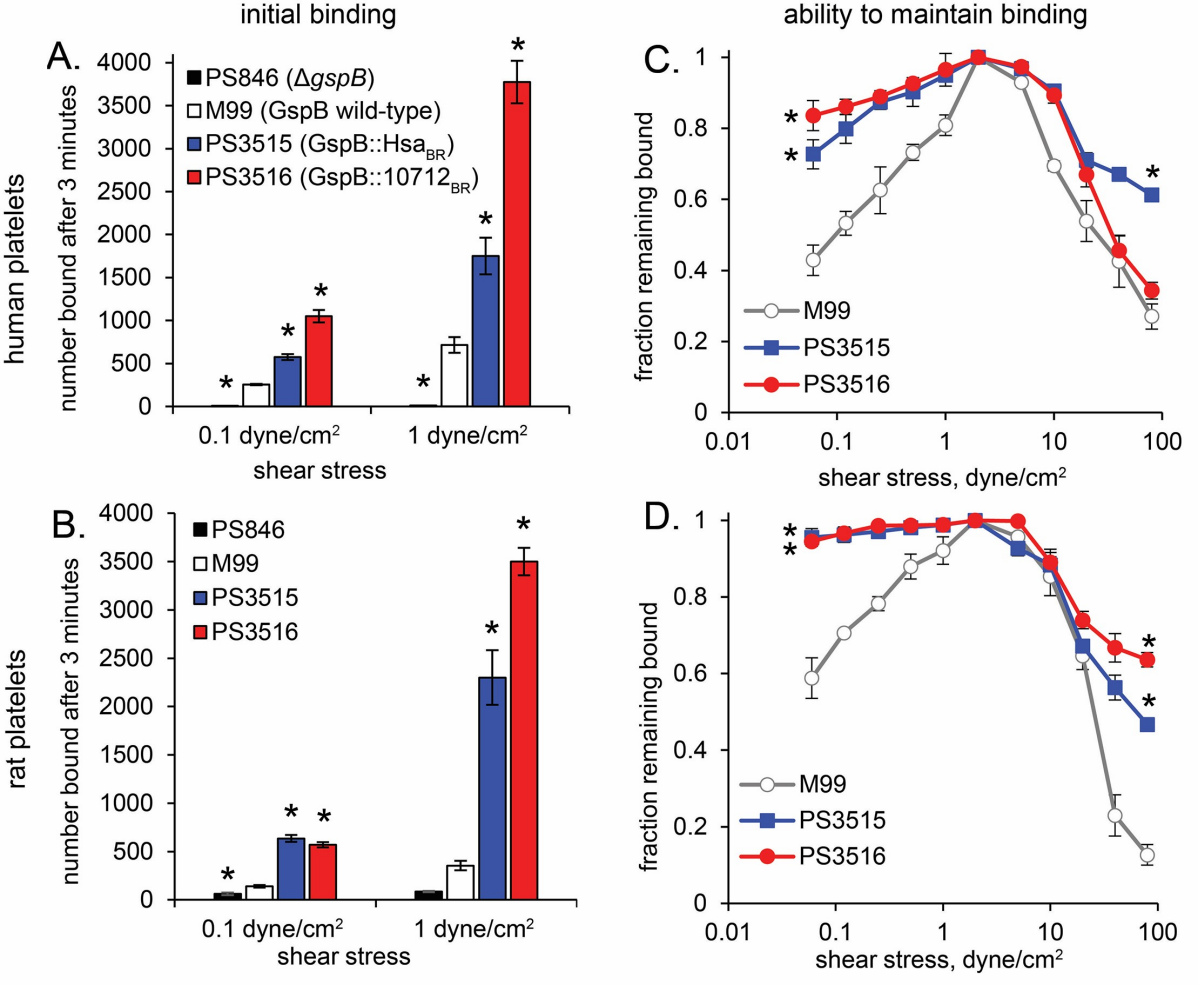
Binding of *S*. *gordonii* M99 and the isogenic variant strains to immobilized human or rat platelets under shear. The *gspB* deletion strain PS846 has previously been used to assess GspB-dependent binding and virulence [15, 26, 30, 36, 49]. Suspensions of bacteria were washed through microfluidic chambers containing immobilized human (A) or rat (B) platelets, at flow rates corresponding to the indicated shear stresses, and adherent bacteria recorded using videomicroscopy and counted. To measure detachment from human (C) or rat (D) platelets, bacteria that initially bound at 1 dyne/cm^2^ were subjected to stepwise lower or higher flow rates, and the fraction of bacteria remaining bound at the indicated shear stresses were counted. Asterisks indicate p<0.05 compared with M99.

A number of studies have linked bacterial binding to platelets with increased virulence in animal models of IE [15, 16, 30, 44–47]. It was therefore surprising that the isogenic variant strain that had the highest level of binding to human platelets *in vitro* (Fig 4C) showed lower binding to rat valves *in vivo*. One possibility was that the isogenic variants might be impaired for binding to rat platelets. In addition, since the SRR adhesins exhibit mechanically activated shear-enhanced adhesion [48, 49], it was conceivable that the isogenic variants could not bind to platelets on valve surfaces, due to the high shear conditions present *in vivo*. To assess these possibilities, we compared binding of the strains to immobilized human and rat platelets under various shear levels. At low shear (0.1 dyne/cm^2^), the strains bound to human platelets similarly to what was seen earlier (Fig 4C), although M99 displayed significantly lower adherence than both PS3515 and PS3516 (Fig 7A). The same relative binding of strains was observed with rat platelets (Fig 7B), with M99 less adherent than the isogenic variants. Binding to human or rat platelets at high shear (1.0 dyne/cm^2^) increased 2- to 4-fold for all strains as compared with binding under low shear. Thus, the lower attachment of PS3515 and PS3516 seen *in vivo* is not likely due to lower binding of these strains to rat platelets, or to differences in shear-enhanced binding.

We also measured the ability of bacteria to remain bound to platelets in variable flow conditions. After allowing the strains to attach under flow at 1 dyne/cm^2^, the shear stress was either decreased to low levels, or increased to the high levels found near the heart valve surfaces (20–80 dyne/cm^2^). In both cases, M99 detached from rat and human platelets at similar or greater levels than did PS3515 and PS3516 (Fig 7C and 7D). Therefore, the lower levels of PS3515 and PS3516 relative to M99 found on aortic valves at 1 h *in vivo* do not reflect a lesser ability of the variant strains to maintain attachment in the very high shear stress of the intracardiac environment. However, the results are consistent with an increased ability for M99 to detach from cardiac valves and disseminate to other organs via the bloodstream.

### Differences between human and rat GPIb sialoglycans

**Fig 8:**
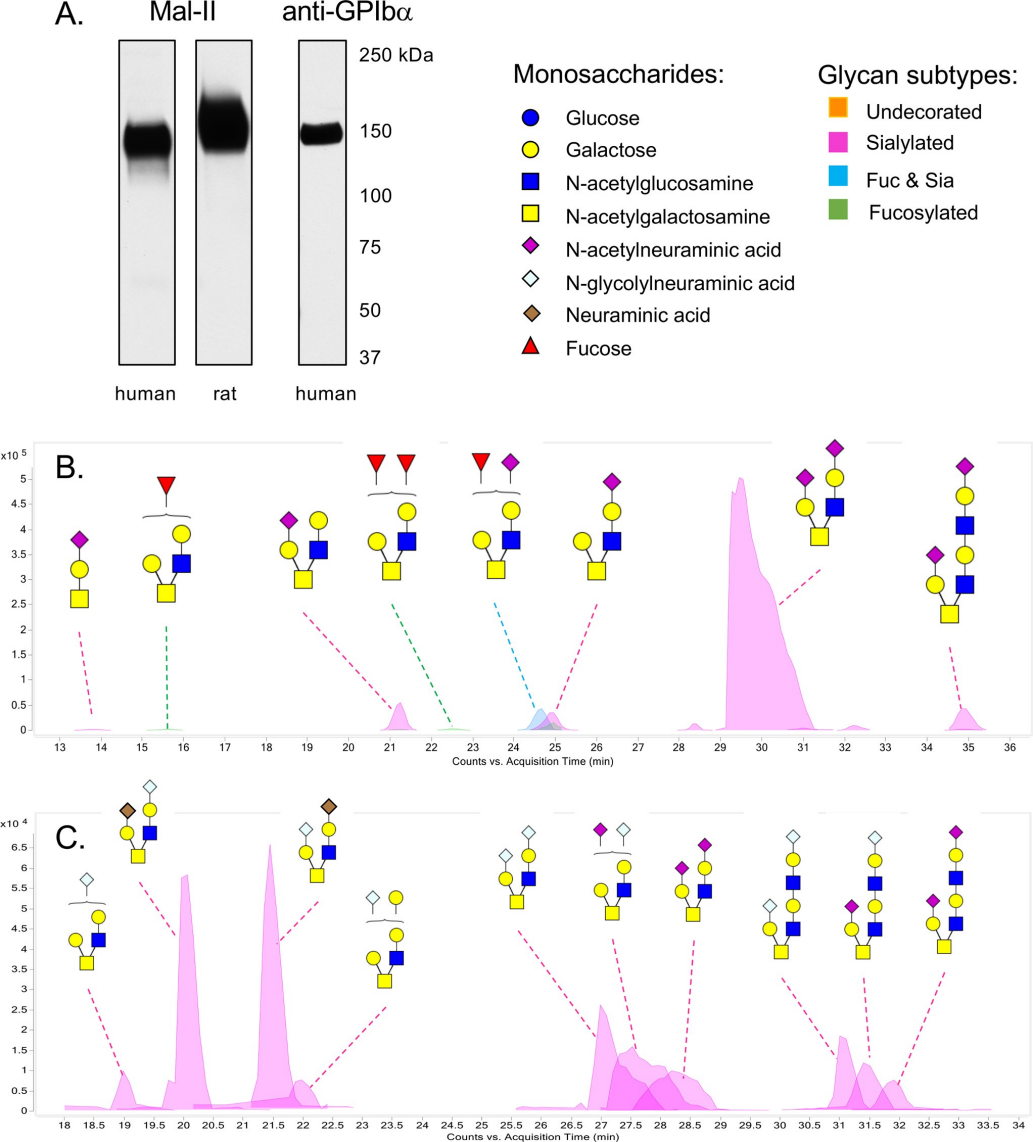
Comparison of human and rat platelet GPIbα O-glycans. A: Western and lectin blot analysis showing GPIbα as the major sialylated glycoprotein in the crude platelet extracts. Lanes contain 2 μl of the GPIbα preparations. Proteins were separated by electrophoresis on 3–8% polyacrylamide, transferred to nitrocellulose, and then probed as indicated. Mal-II is a lectin commonly used to detect α2–3 sialic acids, and is specific for sTa and di-sialylated T-antigen [68]. The anti-GPIbα antibody is specific for the human protein. An antibody that recognizes the rat homolog in western blots is not currently available. B: Putative structures of the O-glycans released from the human GPIbα sample. The structures are based on the precise masses and inferred monosaccharide composition (Table 4) in addition to the MS/MS fragmentation patterns. Brackets indicate cases where the position of monosaccharides could not be assigned. Monosaccharide symbols follow the Symbol Nomenclature for Glycans system [67]. C: Putative structures of the O-glycans released from the rat GPIbα sample. The structures are based on the precise masses and inferred monosaccharide composition (Table 5) in addition to MS/MS fragmentation data. Brackets indicate cases where the position of monosaccharides could not be assigned.

We previously determined that GspB and the 10712_BR_ bind less readily to sialoglycans terminating in the Neu5Gc versus Neu5Ac form of sialic acid, whereas Hsa binds readily to both [23, 26]. We postulated therefore that the slightly lower binding of M99 and PS3516 to rat versus human platelets, at least at low shear, might be due to the presence of Neu5Gc on the former (unlike rats and many other mammals, humans do not produce Neu5Gc [50]). To examine this directly, we assessed the sialic acid composition of GPIbα from rat versus human platelets. We chose to examine a minimally-processed sample, to avoid the loss of labile groups (e.g. O-acetyl) or the unintentional selective enrichment of glycoform sub-populations that can occur during purification. GPIbα was the major sialylated glycoprotein in the crude extracts of both rat and human platelets, as determined by western blotting and by probing the samples with the sialic acid-binding lectin Mal-II (Fig 8A). HPLC of chemically-released sialic acids from both the human and rat GPIbα had minor amounts of O-acetylated sialic acids (contributing to 5% or 13% of the total sialic acids, respectively; Table 3). However, more than half of the sialic acid content of the rat platelet GPIbα extract was Neu5Gc, rather than Neu5Ac. This finding largely explains why M99 and PS3516 showed somewhat lower binding to rat versus human platelets.

**Table 3:** Sialic acid composition of human versus rat platelet GPIbα extract.^a^

	Neu5Gc	Neu5Ac	Neu5Gc8Ac	Neu5,8Ac2	Neu5,9Ac2	Neu4,5Ac2	total
**Human**	n/d	94.91	n/d	2.63	2.46	n/d	100
**Rat**	58.37	28.32	5.37	n/d	4.77	3.17	100

^a^ values represent the percent of total sialic acids; Neu5Gc8Ac, 8-O-acetyl Neu5Gc; Neu5,8Ac2, 8-O-acetyl Neu5Ac; Neu5,9Ac2, 9-O-acetyl Neu5Ac; Neu4,5Ac2, 4-O-acetyl Neu5Ac; n/d not detected

We also examined the O-glycan structures, in order to look for differences in core 1 glycans such as sTa, versus core 2 glycans which typically have sLn branches. We found that a core 2 hexasaccharide constitutes 87% of the total O-glycans in the human GPIbα extract (Fig 8B and Table 4), consistent with earlier reports showing this as the major O-glycan on purified human GPIbα [51, 52]. Also consistent with previous reports, a relatively minor amount of sTa was detected. However, a larger core 2 octasaccharide. rather than di-sialylated core 1 glycan, was evident as an additional minor glycan. In comparison, the rat GPIbα sample had a more heterogenous distribution of O-glycans, largely due to the variety of modified sialic acid forms (Fig 8C and Table 5). In agreement with the total sialic acid analysis, slightly more Neu5Gc than Neu5Ac was evident. An unexpected finding was the presence of neuraminic acid (Neu), in addition to Neu5Ac and Neu5GC, and thus adding to the heterogeneity of O-glycans on rat GPIbα. Di-sialylated core 2 hexasaccharides were still the most abundant structures (although as a mixed population), and di-sialylated core 2 octasaccharides were also evident. Although lectin blotting with Mal-II indicated that sTa was present on rat platelet GPIbα (Fig 8A), the amount was apparently below the level of detection by mass spectrometry. The higher abundance of sLn-bearing core 2 glycans versus sTa (epitopes recognized by Hsa and 10712_BR_ but not GspB) may explain why M99 shows relatively low binding to human and rat platelets, compared with the levels of binding by PS3515 and PS3516. The combined results suggest that streptococcal interaction with a minor O-glycan on GPIbα may be more important than the over-all affinity for GPIbα for pathogenic effects.

**Table 4:** Identification by mass spectrometry of O-glycans released from human platelet GPIbα.

RT (min)^a^	Mass^b^	m/z^c^	Volume	% Total	Composition^d^
**13.818**	676.259	677.264	55954	0.1	1-1-0-1
**15.621**	896.351	897.355	66130	0.2	2-2-1-0
**21.22**	1041.385	1042.393	1109306	2.9	2-2-0-1
**21.423**	1058.403	1059.408	34603	0.1	3-2-1-0
**22.534**	1042.406	1043.414	86869	0.2	2-2-2-0
**24.62**	896.34	897.357	34603	0.1	3-2-1-0
**24.641**	1187.442	594.729	995826	2.6	2-2-1-1
**24.894**	1041.386	1042.393	892163	2.3	2-2-0-1
**24.913**	750.287	751.296	321269	0.8	2-2-0-0
**28.375**	1332.473	667.248	202429	0.5	2-2-0-2
**29.585**	1332.482	667.249	33180240	86.0	2-2-0-2
**31.03**	1697.612	849.818	112008	0.3	3-3-0-2
**32.243**	1332.49	667.253	224937	0.6	2-2-0-2
**34.9**	1697.618	849.815	1199390	3.1	3-3-0-2
**34.904**	1041.396	1042.402	66130	0.2	2-2-0-1

^a^Retention time

^b^Daltons

^c^Precursor ion mass to charge ratio

^d^Hex-HexNAc-Fuc-NeuAc

**Table 5:** Identification by mass spectrometry of O-glycans released from rat platelet GPIbα.

RT (min)^a^	Mass^b^	m/z^c^	Volume	% Total	Composition^d^
**19.004**	1057.379	529.698	195074	3.0	2-2-0-0-1-0
**20.048**	1306.461	654.238	1413851	21.6	2-2-0-1-0-1
**21.463**	1306.465	654.24	1422112	21.7	2-2-0-1-0-1
**21.987**	1219.424	610.722	219467	3.4	3-2-0-0-1-0
**27.08**	1364.468	683.241	895174	13.7	2-2-0-0-2-0
**27.596**	1348.477	675.244	877659	13.4	2-2-0-1-1-0
**28.244**	1332.481	667.249	555317	8.5	2-2-0-2-0-0
**31.083**	1729.599	865.808	406896	6.2	3-3-0-0-2-0
**31.467**	1713.611	857.813	348183	5.3	3-3-0-1-1-0
**31.889**	1697.613	849.817	214646	3.3	3-3-0-2-0-0

^a^Retention time

^b^Daltons

^c^Precursor ion mass to charge ratio

^d^Hex-HexNAc-Fuc-NeuAc-NeuGc-Neu

## Discussion

These studies aimed to determine whether the binding of streptococci to different sialoglycan structures has an impact on the pathogenesis of IE. Our results indicate that there are at least two means by which sialoglycan binding can impact virulence. First, binding to sLn is correlated with a reduced initial colonization of the aortic valves, as compared with sTa binding. Since binding to sLn versus sTa does not appear to promote clearance from the blood, it is likely that binding to one or more sLn-modified targets could divert bacteria away from the damaged endocardium. The potential "off-target" glycans could be displayed on plasma proteins or blood cells. The most likely off-targets for the GspB::10712_BR_ expressed by PS3516 are the core 2 sialoglycans of GPIbα on circulating platelets. Support for core 2 sialoglycan off-targets in blood was seen in a previous study of *S*. *sanguinis* and *S*. *gordonii*, where one or more components of whole blood diverted a SrpA+ *S*. *sanguinis* strain (which has high affinity for platelet GPIbα and core 2 sialoglycans), but not *S*. *gordonii* M99, away from surfaces under conditions of flow [49]. The finding that binding to sLn and core 2 sialoglycans *in vitro* is associated with a negative impact on aortic valve colonization is also consistent with IE studies using *S*. *sanguinis* SK36, in which a Δ*srpA* variant was slightly more virulent than the parental strain [21]. However, whether the Δ*srpA* variant showed increased initial attachment was not determined. It is also likely that initial colonization of aortic valves by *S*. *gordonii* is strongly influenced by other surface adhesins, such as PadA (binding the IIbIIIa fibrinogen receptor on activated platelets), CshA (adherence to fibronectin), and SspA/B [53–57]. The relative contribution of these adhesins has not been assessed *in vivo*, and such studies would benefit from better *in vitro* models of damaged cardiac valve endothelium.

In addition to the negative impact of sLn binding on the initial colonization of aortic valves, sTa binding appears to enhance disease progression. That is, strains that can bind sTa (M99 and PS3515) had two-log higher levels of bacteria (CFU/g) within aortic valve vegetations at 72 h post-infection, compared with the strain that does not bind sTa (PS3516; Fig 5). These differences in bacterial densities are on par with previous assessments of Δ*gspB* or Δ*hsa* strains [15, 16]. The findings confirm that binding to sTa, rather than to sialic acid *per se*, is a virulence property. In addition, the growth of PS3515 to high densities, despite lower initial colonization, indicates that sTa binding contributes to later stages in the formation of macroscopic vegetations. If platelet GPIbα is the key sTa-modified target, sTa-binding adhesins such as GspB and Hsa may play a critical role in the subsequent capture of circulating platelets, or in modulating the aggregation or activation of the captured platelets. Since sTa was confirmed to be a minor glycan on platelet GPIbα, the results suggest that binding to a unique glycosite on GPIbα is important for these events. For example, binding to sT-modified glycosites near the N-terminal leucine-rich repeat domain of GPIbα, which encompasses the binding sites for vWF and thrombin and contributes to dozens of indirect interactions with other clotting factors [58], could have localized effects on properties of the platelet-fibrin thrombus. In turn, this could impact the ability of streptococci to persist within aortic thrombi, thus contributing to the severity of disease. The impact of GspB and Hsa on platelet function and thrombus properties likely occurs in concert with other interactions, especially PadA with platelet IIbIIIa (the fibrinogen receptor) and secreted factors such as Challisin, which has been reported to cleave fibrinogen [44, 54, 59–61].

Aside from the role of sialoglycan binding in pathogenesis, a second question addressed in these studies is whether the rat and human GPIbα O-glycans are similar or different. We found that GPIbα from both species has a disialylated core 2 hexasaccharide as the major O-glycan, but the rat O-glycans display a greater variety of modified sialic acid forms. An unexpected finding was the presence of Neu, in addition to Neu5Ac and Neu5Gc, thus adding to the heterogeneity of O-glycans on rat GPIbα. Possibly due to a mix of Neu, Neu5Ac and Neu5Gc forms, sTa was not detected by mass spectrometry of the rat platelet GPIbα O-glycans. However, the binding of M99 versus the Δ*gspB* strain PS846 to rat platelets (Fig 7), and the strong reactivity seen with the Mal-II (Fig 8A), are strongly indicative of the presence of sTa. Since Hsa can readily bind the Neu5Gc form of sTa [23, 26], this may explain why PS3515 produced high bacterial densities in the aortic valve vegetations 72 hours after infection (Fig 5), despite the lower initial attachment (Fig 6). Based on our aggregate findings, we would predict that a variant of *S*. *gordonii* that is selective for sTa, but that can readily bind both the Neu5Ac and Neu5Gc forms, would be the most virulent in animal models of IE. Future studies will address this question.

An ongoing challenge in determining the precise mechanisms by which sialoglycan binding can drive or attenuate virulence, and whether interactions with sialylated glycoproteins beyond platelet GPIbα contribute to pathogenesis, is the limited knowledge regarding where and when specific O-glycan structures are expressed within the endovascular space. Regarding the role of sTa binding, it is possible that interactions with O-glycosylated proteins other than platelet GPIbα could contribute to streptococcal survival in the infected endocardium. However, the other sTa-modified glycoprotein ligands for Siglec-like adhesins identified thus far (red blood cells and several plasma proteins) are not known components of the aortic valve vegetations. Similarly, for sLn and core 2 glycans as off-targets, it is unknown whether other blood cells display a higher density of sialylated O-glycans than do platelets. Other potentially important off-target glycan ligands not yet specifically addressed, but recognized by the 10712_BR_ and several other Siglec-like BRs, include sulfated or fucosylated derivates of sLn, such as sialyl Lewis X (Fig 2). Although there is little, if any, of these other structures on GPIbα or plasma proteins recognized by the Siglec-like BRs [33], in samples obtained from healthy individuals, it is unknown whether they may be more abundant in conditions of vascular damage or chronic valve disease that occur in susceptible human patient populations. As we continue to hone our understanding of the ligand specificities of the Siglec-like BRs, we can use the recombinant adhesins as probes to monitor spatial and temporal changes in specific sialoglycan epitopes in different human tissues and in the animal models of disease.

## Methods and materials

### Ethics statement

Human blood was collected from volunteers under a protocol approved by the Committee on Human Research at UCSF (IRB number 11–06207) or at the University of Washington (IRB number 29332). All donors provided written informed consent. Animal studies were approved by the Los Angeles Biomedical Research Institute animal use and care committee (IACUC number 31311–01, reference number 044163), and followed the United States Public Health Service Guide for the Use and Care of Laboratory Animals.

### Media and other reagents

Todd-Hewitt broth (THB; Difco Laboratories), or Todd-Hewitt agar (THA) containing 8% (v/v) defibrinated sheep blood (Hardy Diagnostics) were used as bacterial culture media. Spectinomycin (100 μg/ml) or chloramphenicol (15 μg/ml) was added to solid media as indicated. Antibiotics and Dulbecco's phosphate buffered saline (DPBS) were from Sigma.

### *S*. *gordonii* strains and construction of isogenic variants

*S*. *gordonii* M99 is a previously-described strain that was recovered from the blood of an endocarditis patient [62]. Strains PS846 (M99Δ*gspB*) and PS2114 (M99Δ5'*gspB*::*cat*) lack expression of GspB and were described elsewhere [30, 36]. Replacement of the BR region of *gspB* in strain PS2114 was accomplished using a "knock in" strategy (Fig 3) similar to that used for generating point mutations in *gspB* [30]. We initially sought to replace just the Siglec domain of the BR, since this is the only region that contacts sialoglycans. However, when examining recombinant BRs, we found that fusing the Siglec domain to a heterologous Unique domain rendered the chimeric BR prone to degradation when expressed in *E*.*coli*. We learned subsequently that this was likely due to a mis-match at the domain interface, i.e. the inter-domain angle is quite different in Hsa_BR_ or the 10712_BR_ versus GspB_BR_ (manuscript submitted, and see Fig 1). We therefore chose to replace the entire BR as follows. Chimeric sequences spanning codons 222 to 703 of *gspB*, but with the BR coding sequence altered as detailed in Fig 3 and including a 3' NotI restriction site, were synthesized (Life Technologies GeneArt Strings) and used to replace the SalI-NotI fragment spanning codons 231 to 602 of *gspB* in plasmid pS326B602 (pS326 carrying 3'*pdxU*::*spec*::*gspB*^*1-602*^; the SalI restriction site is at *gspB* codon 231). The resulting plasmid was introduced to strain PS2114 by natural transformation. Note that this strategy was designed to force downstream recombination within the ~300 nucleotide stretch of the SRR2 coding region spanning codons 605 to 703, which is substantially different from the remainder of the SRR2 coding region, in order to avoid indiscriminate recombination further downstream and potential alteration of the length of SRR2. Colonies were selected on spectinomycin and scored for loss of chloramphenicol resistance, indicative of double crossover and gene replacement rather than plasmid insertion. To confirm the expected replacement, chromosomal DNA was extracted using the Wizard Genomic DNA Purification Kit (Promega). A 4 kb region spanning 5'*pdxU* to *gspB* codon 1060 was amplified by PCR, and then subjected to DNA sequence analysis (Sequetech). The insertion of *spec* upstream of *gspB* (PS2161) was previously determined not to affect virulence [30].

### *S*. *gordonii* growth rate determination and GspB variant expression

To determine growth rates, strains were grown in THB for 17 h at 37°C, diluted 1:10 into fresh medium and split to 9 × 1 ml in 5 ml snap-cap tubes. The cultures were incubated in a 37°C water bath without shaking, and tubes were removed after 1 to 23 h, vortexed, and the contents transferred to a cuvette to determine the optical density at 600 nm. The experiment was repeated twice, and a representative experiment is shown. To assess surface expression of the GspB variant adhesins, cell wall proteins were extracted with mutanolysin, and proteins were monitored by western blotting with a polyclonal antibody that recognizes the glycan moieties on GspB, as described previously [63].

### Preparation of human and rat platelets

Human platelets were prepared from citrate-anticoagulated blood donated by healthy volunteers as described [62]. Rat platelets were prepared from sodium citrate-treated pooled Sprague-Dawley rat blood (Innovative Research, Novi MI). Prostaglandin I_2_ (Cayman Chemical Company) was added to 1 μg/ml final concentration. Platelet-rich plasma was obtained by centrifugation of whole blood for 15 min at 250 × *g*, followed by 10 min at 500 × *g*. Platelets were separated by centrifugation at 1000 × *g* for 10 min. Platelets were washed twice with 140 mM NaCl, 6 mM dextrose, 1 mM EDTA, 20 mM Hepes pH 6.6, and then either fixed [64] or used for GPIbα extraction as indicated.

### Binding to immobilized platelets or glycans

Washed, formaldehyde-fixed platelets were immobilized in microtiter wells, and the binding of *S*. *gordonii* was determined as described [63]. To assess binding to immobilized synthetic glycans, biotinylated glycans (Glycotech) were immobilized in NeutrAvidin-coated microtiter wells (Thermo Scientific). After incubating 1 h at RT, wells were rinsed twice with DPBS to remove any unbound glycans. Wells were blocked with 50 μl of a Blocking Reagent (Roche) 1X in DPBS. Excess block was removed, and 50 μl of *S*. *gordonii* strains that had been grown 17 h in THB, washed twice in DPBS, sonicated briefly to disrupt any chains, and then suspend at ~5 x 10^8^ per ml in DPBS were added. Plates were incubated for 1.5 h at RT with moderately vigorous mixing on a rotational platform, and any unbound bacteria were removed by aspiration and washing the wells twice with 100 μl DPBS. Bound bacteria were released by adding 50 μl of a trypsin solution (1 mg/ml DPBS), incubating 30 min at 37°C followed by 30 min at RT, and then plating dilutions on sheep blood agar to enumerate the percent of the inoculum bound. Differences between means were compared for statistical significance using a one-way ANOVA, followed by the Sidak's multiple comparisons test, and using p≤0.05 as the threshold for significance.

### Binding to immobilized human or rat platelets under shear

*S*. *gordonii* binding to immobilized human or rat platelets under shear, using microfluidic flow chambers (GlycoTech), was performed as described previously for human platelets [49] and using rat platelets prepared as described above. Differences in the binding of M99 versus each of the variant strains were assessed by comparing the means for statistical significance using a one-way ANOVA, followed by the Dunnett's multiple comparisons test, using p≤0.05 as the threshold for significance. Differences in detachment were assessed only at the lowest and highest shear levels.

### Rat model of endocarditis

Infective endocarditis was produced in Sprague-Dawley female rats (250–300 g; Envigo) as described previously [15], with the following modifications. Animals were anesthetized with ketamine (35 mg/kg) and xylazine (10 mg/kg). A sterile polyethylene catheter was surgically placed across the aortic valve of each animal, such that the tip was positioned in the left ventricle, and left in place throughout the experiment. Three days post-catheterization rats were infected IV with an inoculum of either 1 x 10^5^ CFU of single *S*. *gordonii* strains, or with 2 x 10^5^ CFU of a pair of strains at a 1:1 ratio, as indicated. At 72 h post-infection, animals were sacrificed with pentobarbital (200 mg/kg, intraperitoneally). All cardiac vegetations, as well as samples of the kidneys and spleens, were harvested, weighed, homogenized in saline, serially diluted, and plated onto THA to determine the number of bacteria in the homogenized tissues. For the competition studies, bacterial colonies were plated onto THA and THA containing spectinomycin, in order to determine the CFU/g of M99 and the isogenic variant strain. The number of bacteria within tissues was expressed as the log_10_ CFU per gram of tissue. Differences between means were compared for statistical significance using a paired *t-*test (for competition studies), or by one-way ANOVA, followed by the Tukey correction for multiple comparisons (for single strain infections), using p≤0.05 as the threshold for significance.

Differences in the initial *in vivo* adherence of these strains to the endocardium were assessed using the single strain infection model described above, except that rats were infected with either 10^8^ or 10^7^ CFU (levels determined to be at or above the level of detection, and below the level of saturated binding). At 1 h post-infection, blood samples were obtained, animals were sacrificed and the cardiac vegetations harvested for quantitative culture.

### Preparation of platelet GPIbα extracts

A crude extract containing platelet GPIbα was prepared using the method of Korrel et al [52], with the following modifications. Washed platelets, obtained from 25 ml of healthy human donor blood or pooled rat blood as described above, were suspended in 1.5 ml DPBS supplemented with 2 mM CaCl_2_. The platelet suspension was sonicated for 15 sec, and then incubated at 37°C for 1 h. Cellular debris was removed by centrifugation at 16,000 × *g*, and the GPIbα-containing supernatant was filtered through a 0.45 μm membrane, and then stored at -20°C.

### Western and lectin blotting of platelet GPIbα

Human or rat platelet extracts were combined with LDS sample buffer (Invitrogen) and dithiothreitol (50 mM final concentration), boiled for 5 min, and then loaded to wells of a 3–8% polyarylamide gradient gel (Invitrogen). Following separation by electrophoresis, proteins were transferred to BioTraceNT (Pall Corporation) and then probed via western blotting with anti-GPIbα (Abcam anti-CD42b) or via lectin blotting with Mal-II (Vector Laboratories) as described [33].

### Identification and quantitation of sialic acid content of the GPIbα extracts

Sialic acids were released from platelet GPIbα by treating the extract with acetic acid (2N final concentration) at 80°C for 3 h, filtered through a 10kD centrifugal filter (Microcon), and dried using a vacuum concentrator (SpeedVac). The released sialic acids were labeled with 1,2-diamino-4, 5-methylenedioxybenzene (DMB, Sigma Aldrich) for 2.5 h at 50°C [65]. HPLC analysis was performed using a Dionex UltiMate 3000 system with an Acclaim C18 column (ThermoFisher) under isocratic elution in 7% methanol, 7% acetonitrile, and 86% water. Sialic acid standards were derived from commercially available bovine submaxillary mucin, Neu5Gc and Neu5Ac (Sigma Aldrich) as well as from normal horse serum.

### GPIbα O-glycan profiling

The analysis of O-glycans was performed on the same GPIbα extract used for sialic acid analysis. The glycoprotein sample was suspended in 5 mM dithiothreitol in 100 mM ammonium bicarbonate buffer (pH = 7.5) and denatured by heating in boiling water for 1 min. The N-glycans were released from the protein by digestion with peptide:N-glycosidase F (PNGase F, New England Biolabs), and the de-N-glycosylated proteins were precipitated with chilled ethanol. The O-glycans were chemically released via beta elimination by resuspending the precipitated proteins in 1 M sodium borohydride and 0.1 M sodium hydroxide. After 18 h at 45°C, the reaction was quenched with acetic acid. The released O-glycans were purified using solid phase extraction with porous graphitic carbon and hydrophilic interaction liquid chromatography. Glycan samples were analyzed on an Agilent 6520 Accurate Mass Q-TOF LC/MS equipped with a porous graphitic carbon microfluidic chip. A binary gradient consisting of (A) 0.1% formic acid in 3% acetonitrile, and (B) 1% formic acid in 89% acetonitrile was used to separate the glycans at a flow rate of 0.3 μl/min. Data were processed with Agilent MassHunter B.07 software, using the Find by Molecular Feature algorithm with an in-house library of O-glycan masses and chemical formulae to identify and quantitate the O-glycan signals.

## Supporting information

S1 Fig Coomassie stain and uncropped Western blot and of cell wall proteins extracted from *S*. *gordonii* M99 and isogenic variant strains. Lanes contain cell wall proteins extracted from bacteria in 75 μl of stationary-phase cultures cultures (roughly 7.5 x 10^7^ CFU; lanes 1–4) or, to enhance visibility of the proteins, from 200 μl of stationary-phase cultures (roughly 2 x 10^8^ CFU; lanes 5–8). Gels were either stained with Coomassie (left panel) or transferred to nitrocellulose and probed with polyclonal antibodies that recognize the glycan moieties on GspB (right panel). Lanes 1 and 5, the Δ5'*gspB* strain PS2114 (no GspB expressed); lanes 2 and 6, M99 (GspB wild-type); lanes 3 and 7, PS3515 (GspB::Hsa_BR_); lanes 4 and 9, PS3516 (GspB::10712_BR_); lanes marked "M" contain molecular weight markers (250, 150, 100, 75, 50 and 37 kDa from top to bottom).
(TIF)
