# Supplementary figures and images for: Recognition of specific sialoglycan structures by oral streptococci impacts the severity of endocardial infection
Source: PLoS Pathog. 2019 Jun 24;15(6):e1007896. doi: 10.1371/journal.ppat.1007896 (PMC6611644; doi:10.1371/journal.ppat.1007896)

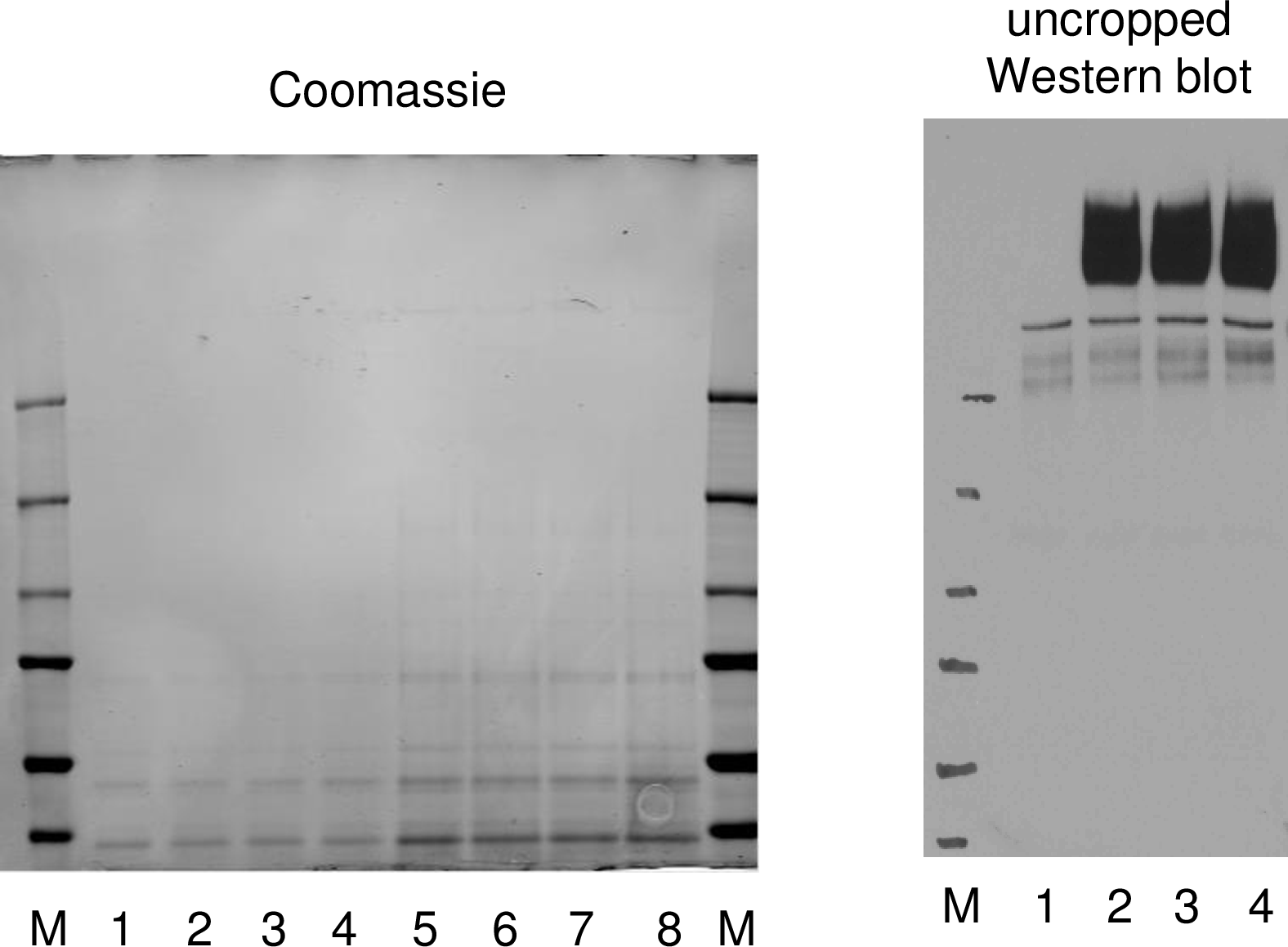

Supplement: S1 Fig — Lanes contain cell wall proteins extracted from bacteria in 75 μl of stationary-phase cultures cultures (roughly 7.5 x 10^7^ CFU; lanes 1–4) or, to enhance visibility of the proteins, from 200 μl of stationary-phase cultures (roughly 2 x 10^8^ CFU; lanes 5–8). Gels were either stained with Coomassie (left panel) or transferred to nitrocellulose and probed with polyclonal antibodies that recognize the glycan moieties on GspB (right panel). Lanes 1 and 5, the Δ5'gspB strain PS2114 (no GspB expressed); lanes 2 and 6, M99 (GspB wild-type); lanes 3 and 7, PS3515 (GspB::HsaBR); lanes 4 and 9, PS3516 (GspB::10712BR); lanes marked "M" contain molecular weight markers (250, 150, 100, 75, 50 and 37 kDa from top to bottom). (TIF) [file ppat.1007896.s001.tif]
